# Sustained Release of MiR-217 Inhibitor by Nanoparticles Facilitates MSC-Mediated Attenuation of Neointimal Hyperplasia After Vascular Injury

**DOI:** 10.3389/fcvm.2021.739107

**Published:** 2021-10-11

**Authors:** Hong Yu, Yutao Hua, Yecheng He, Yin Wang, Xingjian Hu, Si Chen, Junwei Liu, Junjie Yang, Huadong Li

**Affiliations:** ^1^Department of Otorhinolaryngology, Tongji Medical College, Union Hospital, Huazhong University of Science and Technology, Wuhan, China; ^2^Department of Medicine, University of Alabama at Birmingham, Birmingham, AL, United States; ^3^Department of Clinical Medicine, Suzhou Vocational Health College, Suzhou, China; ^4^Department of Cardiovascular Surgery, Tongji Medical College, Union Hospital, Huazhong University of Science and Technology, Wuhan, China; ^5^Department of Anesthesiology, University of Maryland School of Medicine, Baltimore, MD, United States

**Keywords:** mesenchymal stem cells, atherosclerosis, miR-217, PLGA nanoparticle, vascular injury

## Abstract

Mesenchymal stem cells (MSCs) have been proven capable of differentiating into endothelial cells (ECs) and increasing vascular density in mouse ischemia models. However, the therapeutic potential of MSCs in neointimal hyperplasia after vascular injury is still not fully understood. In this study, we proposed that sustained release of miR-217 inhibitor encapsulated by nanoparticles in MSCs can enhance the therapeutic effects of MSCs on alleviating neointimal hyperplasia in a standard mouse wire injury model. We intravenously administered MSCs to mice with injured arteries and examined neointimal proliferation, endothelial differentiation and senescence. We demonstrated that MSCs localized to the luminal surface of the injured artery within 24 h after injection and subsequently differentiated into endothelial cells, inhibited neointimal proliferation and migration of vascular smooth muscle cells. Transfection of MSCs with poly lactic-co-glycolic acid nanoparticles (PLGA-NP) encapsulating an miR-217 agomir abolished endothelial differentiation as well as the therapeutic effect of MSCs. On the contrary, silencing of endogenous miR-217 improved the therapeutic efficacy of MSCs. Our study provides a new strategy of augmenting the therapeutic potency of MSCs in treatment of vascular injury.

## Introduction

Percutaneous transluminal angioplasty (PTA) and hypertension, are characterized by blood vessel wall thickening and stenosis due to endothelium injury, vascular smooth muscle cell (VSMC) proliferation, and extracellular matrix deposition in the blood vessels. Intimal hyperplasia (IH) manifested by thickening of the tunica intima of a blood vessel is closely related to vascular remodeling diseases. IH after vascular injury is the main cause of postoperative stenosis/occlusion after endovascular treatment and has long been the focus of vascular research. Whether a damage of the vascular surface reforms the endothelium depends on the extent and the duration of the vascular damage (1, 2). Damage to the integrity of vascular endothelial cells (ECs) induces thrombosis and proliferation of VSMCs within the vessels. EC injury induces platelet adhesion, T-lymphocyte, monocyte and macrophage infiltration as well as release of extracellular matrix-degrading enzymes (3, 4). Platelets, ECs and VSMCs generate growth factors, such as platelet derived growth factor (PDGF), fibroblast growth factor (FGF) and epidermal growth factor (EGF), chemotactic substances and mitogens, and stimulate migration of VSMCs to the intima, where the VSMCs and fibroblasts secrete large amounts of extracellular matrix (ECM) at the vascular injury site (5–7). The secreted ECM is continuously deposited at the injury site, resulting in gradual thickening of the intima and eventually stenosis.

Many studies have been performed using different cell types as therapeutics to treat vascular injury-induced IH (8–12). Mesenchymal stem cells (MSCs) have been considered as an effective treatment option for vascular injury owing to their self-renewal and multilineage differentiation capabilities as well as paracrine property (13). Besides, MSCs have important advantages including their easy isolation (from different sources) and preservation without raising any ethical concerns as well as a limited risk of tumorigenesis (14, 15). MSCs can differentiate into ECs and SMCs *in vitro* in the presence of various growth factors (16, 17) and can enhance angiogenesis in a mouse hindlimb ischemia model (18, 19). However, the therapeutic effect of those cells is limited due to their low engraftment rate and their therapeutic potential in neointimal hyperplasia after vascular injury is still not fully understood.

MicroRNAs modulate gene expression on post-transcriptional level and are important regulators of angiogenesis and vascular repair. MiR-217 is one of the microRNAs that play a critical role in regulating angiogenesis. MiR-217 regulates endothelial cell aging through silent information regulator 1 (Sirt-1), which is highly expressed in pluripotent stem cells and plays a vital role in controlling the homeostasis of vascular endothelial cells and angiogenesis (20). However, the half-life of miRNAs is relatively short, and can be quickly degraded by nucleases *in vivo*, thereby limiting its blood stability (19). Promisingly, polylactic acid-glycolic acid (PLGA) is a polymer that has been approved by the U.S. Food and Drug Administration (FDA) for medical applications. A large number of experiments have proved that PLGA-based nanoparticles (NPs) can be used as a delivery system for many therapeutic agents, such as anti-inflammatory drugs, antioxidants, growth factors, antibiotics and therapeutic transgenes (21). In our study, we will use PLGA nanoparticles to encapsulate and control a sustained release of miR-217 inhibitor from MSCs in order to enhance the therapeutic potentials of MSCs in vascular injury of a mouse model.

## Materials and Methods

### Animals

C57BL/6J (8–10 weeks old) mice were purchased from Shanghai Experimental Animal Center (Chinese Academy of Sciences, China). All procedures were performed in accordance with the institutional guidelines and were approved by the Ethics Committee of Wuhan Union Hospital.

### Culturing of MSCs

MSCs were isolated from the bone marrow of C57BL/6J mice and cultured in standard medium with 500 mL a-MEM media (Invitrogen), 50 mL FBS, 5 mL Pen/Strep solution (100X), 5 mL L-glutamine (100X) at 37°C in a humidified 5% CO_2_ atmosphere. The culture medium was replaced every 3 days to remove non-adherent cells. MSCs were analyzed for the expression of the cell surface markers by a flow cytometry assay.

### Identification of Implanted MSCs

GFP-labeled MSCs were transplanted by the tail vein injection. The mice were anesthetized 24 h after cell transplantation and received an ice-cold PBS perfusion. The femoral arteries were immediately harvested and fixed with 4% paraformaldehyde for 10 min. The vessel was split open and put on a slide with the endothelial surface facing upwards and covered with a cover slip following addition of a drop of antifade mounting medium (Beyotime, China). The implanted cells were identified by laser scanning confocal microscope (Leica, Germany).

### Differentiation of MSCs Into ECs

EC differentiation was performed as previously described (19). Briefly, MSCs were cultured in the EC medium containing ascorbic acid, heparin, 2% FBS and some growth factors including 50 ng/mL VEGF, 10 ng/mL bFGF, 20 ng/mL IGF and 5 ng/mL EGF (PeproTech, USA). The inducing medium was changed every 2 days. The differentiation process was closely monitored with cell morphology. Immunofluorescence and flow cytometry were performed to confirm the endothelial phenotype after 10 days of culture.

### PLGA-Based NPs Preparation

PLGA-NPs were generated by an emulsification-solvent evaporation technique under constant magnetic stirring at room temperature using 1.2 mL of agomir aqueous solution (0.84 mg/mL) added to 3 mL of PLGA solution (2 mg/mL, the molar ratio of PLGA to agomir was 6:1) to achieve spontaneous formation of nanoparticles followed by incubation at room temperature for 30 min. The nanoparticles were collected by centrifugation at 13,000 rpm for 10 min, after which the supernatant was discarded, the nanoparticles were resuspended in distilled water, and the packaging efficiency of nanoparticle-agomir miRNA complexes was determined. In order to fully combine PLGA with agomir (nanoparticles/agomir), 3 uL of agomir (19.95 ug/ul) were first added to the sodium tripolyphosphate (TPP) solution (1.2 mL, 0.84 mg/mL) with or without 3 uL of Lipofectamine 2000 and then the mixture was added dropwise to the PLGA solution (3 mL, 2 mg/mL) under constant stirring at room temperature.

### Transfection of MSCs With miRNA or Small Interfering RNA

After 3 days of culture MSCs were re-plated in six-well plates (Corning, USA) at a density of 2.5 × 10^5^ cells/well in the presence of 100 nM of microRNA or siRNA and incubated overnight. For gene knockdown or overexpression miR-217 agomir or miR-217 antagomir, Sirt-1 siRNA or 100 nM of control siRNA (conR) (Ribobio Co., China) were transfected into MSCs, respectively. All siRNAs and miRNAs were transfected into MSCs with Lipofectamine 2000 (Invitrogen, USA) according to the manufacturer's protocol, and the cells were harvested for further analysis 48 h post transfection.

### miRNA Isolation and Quantitative RT-PCR

Total RNA was extracted using a Trizol reagent (Invitrogen, USA) according to the manufacturer's instructions. Reverse transcription was performed by using a SuperScript Reverse Transcriptase (Fermentas, Glen Burnie, MD) and a miR-217 stem loop primer (Sunny Biotechnology Co., China). Real-time PCR quantification of miR-217 was carried out by using SYBR Green PCR master mix (BIO-RAD, CA). All samples were analyzed by a Bio-Rad real-time analyzer (Bio-Rad Laboratories, Hercules, CA). The U6 small nucleolar RNA was used as a housekeeping/small RNA reference gene. The relative gene expression values were obtained after normalization to U6 small nucleolar RNA expression levels.

### Surgical Procedures

C57BL/6 male mice (8–10 weeks old) were anesthetized by intraperitoneal injection of ketamine (1.5 mg/kg) and xylazine (0.3 mg/kg). The groin skin on the right side was depilated and sterilized with 75% alcohol. A groin incision was made under a surgical microscope (Yihua, China). The femoral artery was temporarily tightened with a line at the level of the inguinal ligament, and an arteriotomy was made distal to the epigastric branch. A 0.38-mm flexible angioplasty guidewire (COOK, USA) was then inserted under the help of a micro probe (F.S.T, Germany), the line removed, and the wire advanced to the level of the aortic bifurcation and pulled back with 3 passes. After removal of the wire, the arteriotomy site was sutured. After wounding, mice were divided randomly into 6 groups, and underwent intravenous tail injection with PBS containing 2 × 10^5^ MSCs, MSCs transfected with control siRNA (MSC^CoR^), miR-217 agomir (MSC^miR−217^), miR-217 antagomir (MSC^miR−217I^), Sirt1 siRNA (MSC^Sirt1−I^), respectively. Injured arteries were harvested at 3 weeks with 9 mice in each group.

### Western Blot Analysis

Western blot was performed as described previously (22). Antibodies to Sirt-1 (1:1,000, Proteintech, USA), β-actin (1:1,000, Cell Signaling, USA), HRP conjugated Donkey Anti-Rabbit IgG (1:2,000, Jackson ImmunoResearch, USA) were used for the corresponding protein detection. Protein band densities were assessed by using ImageJ 1.45S digital analysis software (National Institute of Health, USA).

### Senescence-Associated β-Galactosidase Staining

The MSCs were cultured for 3 days in 500 mL a-MEM media (Invitrogen), 50 mL FBS, 5 mL Pen/Strep solution (100X), 5 mL L-glutamine (100X) at 37°C in a humidified 5% CO_2_ atmosphere. Senescence-associated β-galactosidase (SA-β-Gal) staining was performed by using an SA-β-Gal staining kit (BioVision, Milpitas, CA), as described previously (23). Each experiment was repeated 3 times.

### Embryoid Body Generation and Culture

The cells (5 × 10^4^ cells/well) were dispensed in embryoid body (EB) medium onto ultra-low attachment 6-well plates (JET BIOFIL, China) and cultured for 72 h. The EB medium included 100 mL DMEM with 2 mM L-glutamine and without ribonucleosides and ribonucleotides (GIBCO, cat. no. 12800-116), 100 mg/mL penicillin and streptomycin, 3.7 g/L NaHCO3 and 15% of FBS.

### Immunofluorescence

Fixation of tissues was performed with 4% paraformaldehyde in PBS for 20 min and the vessels were processed for OCT (Tissue-Tek, USA) embedding. Five μm-frozen sections were cut with a cryostat, blocked with donkey serum (BD Pharmingen, USA), and incubated in rabbit anti-mouse CD31 (1:50, Abcam, UK) and mouse anti-mouse GFP (1:50, Santa Cruz, USA) overnight at 4°C. Incubation with Cy3-conjugated donkey anti-rabbit and FITC-conjugated donkey anti-mouse secondary antibodies was carried out for 1 h (1:200, Jackson ImmunoResearch, USA) at 37°C. The slides were then treated with DAPI for 10 min at room temperature. The slides were cover-slipped and imaged with a Leica immunofluorescence microscope.

### Immunohistochemistry

Immunocytochemistry was performed according to standard protocols. Slides were pre-incubated with 5% normal donkey serum (Jackson, USA) for 40 min each. The anti-mouse CD31 (1:50, Abcam, USA) antibody was applied at 4°C overnight, followed by the appropriate HRP-conjugated IgG (1:200, Jackson). Sections were counterstained with hematoxylin.

### Cell Adhesion Assay

An MSCs adhesion assay with an endothelial monolayer was carried out according to the procedures described previously with some modifications (24). Mouse brain microvascular endothelial cells (bEnd.3) were seeded onto a 96-well plate to form confluent monolayers. Then bEnd.3 cells were preincubated with 10 U/mL of monocyte chemotactic protein 1 (MCP1, R&D systems, USA) for 4 h. Subsequently, 1 × 10^5^ MSCs labeled with PKH26 (Sigma, USA) were added to the endothelial cell monolayer at 37°C for 1 h. Then non-adherent cells were removed by a gentle wash with PBS for three times. Adherent MSCs were fixed, visualized by fluorescence microscopy and counted in five randomly chosen fields-of-view (FOV) for each well. Each experiment was repeated 3 times.

### Flow Cytometry

The cells were harvested by 0.05% trypsin treatment for 3 min, then the cells were washed once with FACS buffer (0.5% BSA/PBS) and fixed with 1% PFA at 37°C for 10 min followed by washing 3 times. The CD31-PE (1:100, BD), CD144-FITC (1:100, Bioss, China) antibodies were diluted in FACS buffer and incubated with the cells at 4°C for 30 min. After 3 times washing with FACS buffer, the cells were analyzed on the Cytoflex LX flow cytometer (Beckman, USA).

### Analysis of Intracellular ROS Level

Intracellular ROS levels were determined by the fluorescence analysis of cells stained with 5-(and-6)-chloromethyl-2',7'-dichlorodihydrofluorescein diacetate acetyl ester (CM-H_2_DCFDA) (Invitrogen). After treatment with PBS of different miRNAs for 48 hr, MSC were incubated with CM-H_2_DCFDA for 30 min at 37°C followed by three washes with PBS. Then the cells were fixed with 4% paraformaldehyde for 10 min and all images were captured under the fluorescent microscope, and the fluorescence was quantified by Image J Software (National Institute of Health, USA).

### MSC-EC Tube Formation Assay

MSC-EC were firstly transfected with different miRNAs for 48 hr, then tube formation was performed by seeding MSC-EC (2.5 × 10^4^ cells/well) on Matrigel (65 μL/well) into a 96-well plate for 24 h in the incubator. Afterwards, five pictures per well were taken using an Olympus IX83 microscope. The tube length was quantified by Image J Software (NIH).

### Statistical Analyses

Data are expressed as mean ± SD. For experiments including multiple comparisons, *P*-values refer to one-way ANOVA followed by Tukey's honestly significant difference (HSD) test. For experiments including only one comparison, *P*-values refer to unpaired 2-tailed Student's *t*-test. *P* ≤ 0.05 was considered statistically significant. Analyses were performed using Prism 7 software (GraphPad).

## Results

### Nanoparticle-Encapsulated MiR-217 Is Slowly Released

**Figure 1:**
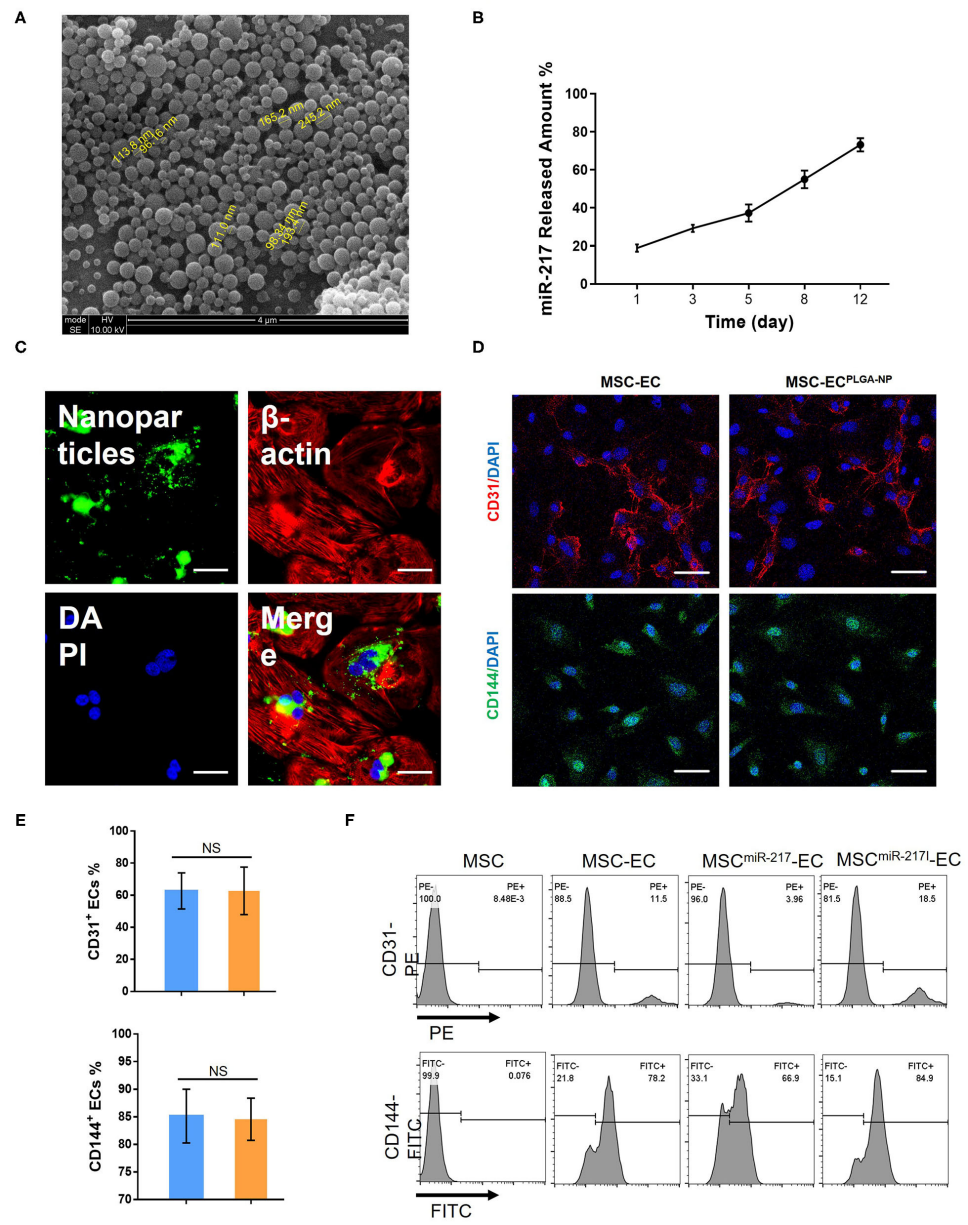
Characterization of PLGA-NP and evaluation of miR-217 effect on endothelial cells (EC) differentiation from MSC. **(A)** PLGA-NP image under a scanning electron microscopy. **(B)** The cumulative amount of miR-217 released from the PLGA-NP was determined *via* qPCR. **(C)** Representative image of MSC after 12 h of incubation with nanoparticles containing green fluorescence dye FAM-labeled control siRNAs. Scale bars = 50 μm. **(D)** Fluorescent imaging of differentiated endothelial cells (EC) from MSC after transfection with or without PLGA-NP. Scale bars = 50 μm. **(E)** Quantification of CD31^+^ and CD144^+^ ECs differentiated from MSC and MSC transfected with NP. **(F)** Flow cytometric analysis of CD31 and CD144 expression on MSC and EC derived from MSC, MSC transfected with agomiR-217 (MSC^miR−217^) or MSC transfected with antagomiR-217 (MSC^miR−217I^).

PLGA nanoparticles (NP) with a mean diameter of 120 nm (Figure 1A) were generated by a double emulsion technique. When 1 mg of nanoparticles loaded with miR-217 agomir was incubated in 1 mL of PBS at 37°C, 20% of the encapsulated miR was released during the first 3 days and 80% was continuously released until day 12 (Figure 1B). Green fluorescence dye FAM-labeled control siRNAs were encapsulated into NPs (Figure 1C green panel), and a large amount of green fluorescent NPs was taken into MSCs from the culture medium within 12 h (Figure 1C).

### MiR-217 Can Modulate the Differentiation MSCs Toward ECs

Internalizaiton of empty NPs did not affect the EC differentiation efficiency as shown by the percentages of differentiated CD31^+^ and CD144^+^ cells from MSCs (Figures 1D,E). However, when miR-217 agomir loaded NPs were internalized by MSCs (MSC^miR−217^), the differentiation efficiency was reduced; on the contrary, miR-217 inhibition (MSC^miR−217I^) improved EC differentiation efficiency (Figure 1F).

### Intravenously Administered MSCs Home to Femoral Artery Injury Sites

**Figure 2:**
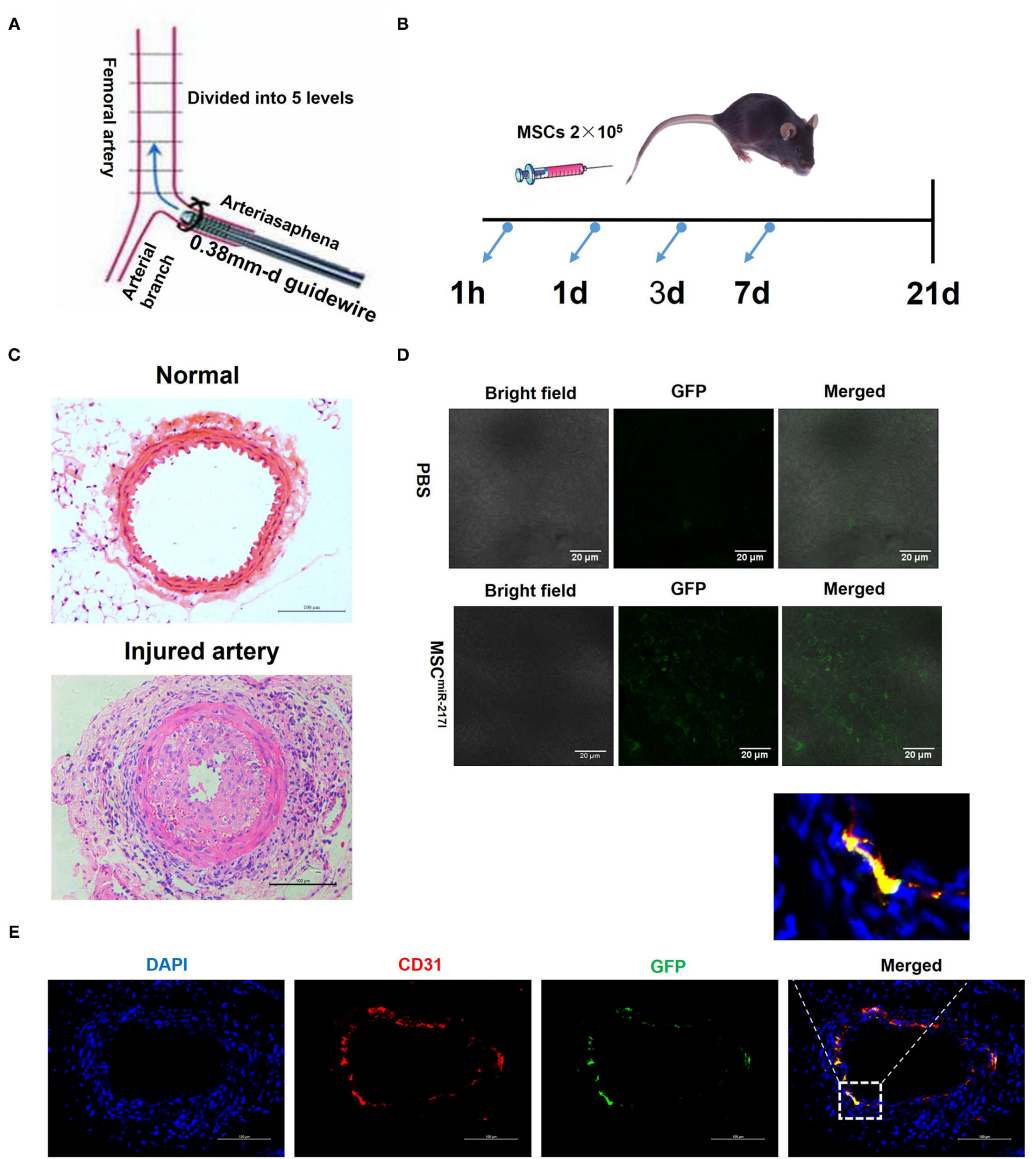
A femoral artery injury model in mice. **(A)** A schematic representation of the surgical procedure. **(B)** A schematic representation about the cell treatment procedure after the femoral artery injury. **(C)** A representative image of uninjured femoral artery and neointima formation 21 days after endothelial cell damage. Scale bars = 100 μm. **(D)** MSCs home to the vascular injury site at 24 h after injection; 2 × 10^5^ GFP-labeled MSCs were intravenously infused directly in the mouse tail vein following the femoral artery injury, and 24 h later the presence of GFP-labeled cells was analyzed by fluorescence microscopy. Representative confocal microscopy images show that the injected MSCs localized exclusively to the site of injury. Scale bars = 20 μm. **(E)** Immunofluorescent analysis revealed differentiation of engrafted GFP-labeled MSC^miR−217I^ into endothelial cells at day 21 after cells injection.

In order to study the therapeutic effect of MSCs on vascular endothelium injury, we first established the standard wire-induced femoral artery injury model using 8–10-weeks-old C57BL/6J mice as previously described (20) (Figure 2A). The cell injection time points were determined at 1 h, day 1, day 3, and day 7 after surgery (Figure 2B). As shown in Figure 2C, the femoral artery of the standard wire-operated C57BL/6J mice showed significant intimal hyperplasia (*n* = 3), as compared to the uninjured femoral artery. To evaluate the therapeutic effects of MSCs on arterial injury, we next injected MSCs (2 × 10^5^ cells) in 200 uL *via* the tail vein. Injecting the same amount of PBS alone served as negative controls. To track the injected MSCs in the recipient mice with femoral artery injury, we used an MSC line stably expressing GFP (GFP-MSCs) and examined the cells using confocal microscopy 24 h after injection. The injured femoral arteries were dissected, and the luminal surfaces were examined. GFP-MSCs were identified on the luminal surface of injured femoral arteries, indicating that MSCs could home to the injury sites (Figure 2D). Three weeks after cell injection, mice were sacrificed, and the femoral arteries were harvested for further analysis. We found that only MSCs treated with NPs encapsulating miR-217 antagomir retained in the injured artery and differentiated into ECs (Figure 2E).

### Intravenously-Administered MSCs Is Sufficient to Exert a Protective Effect on Injured Arteries, Which Is Enhanced by MiR-217 Inhibition

**Figure 3:**
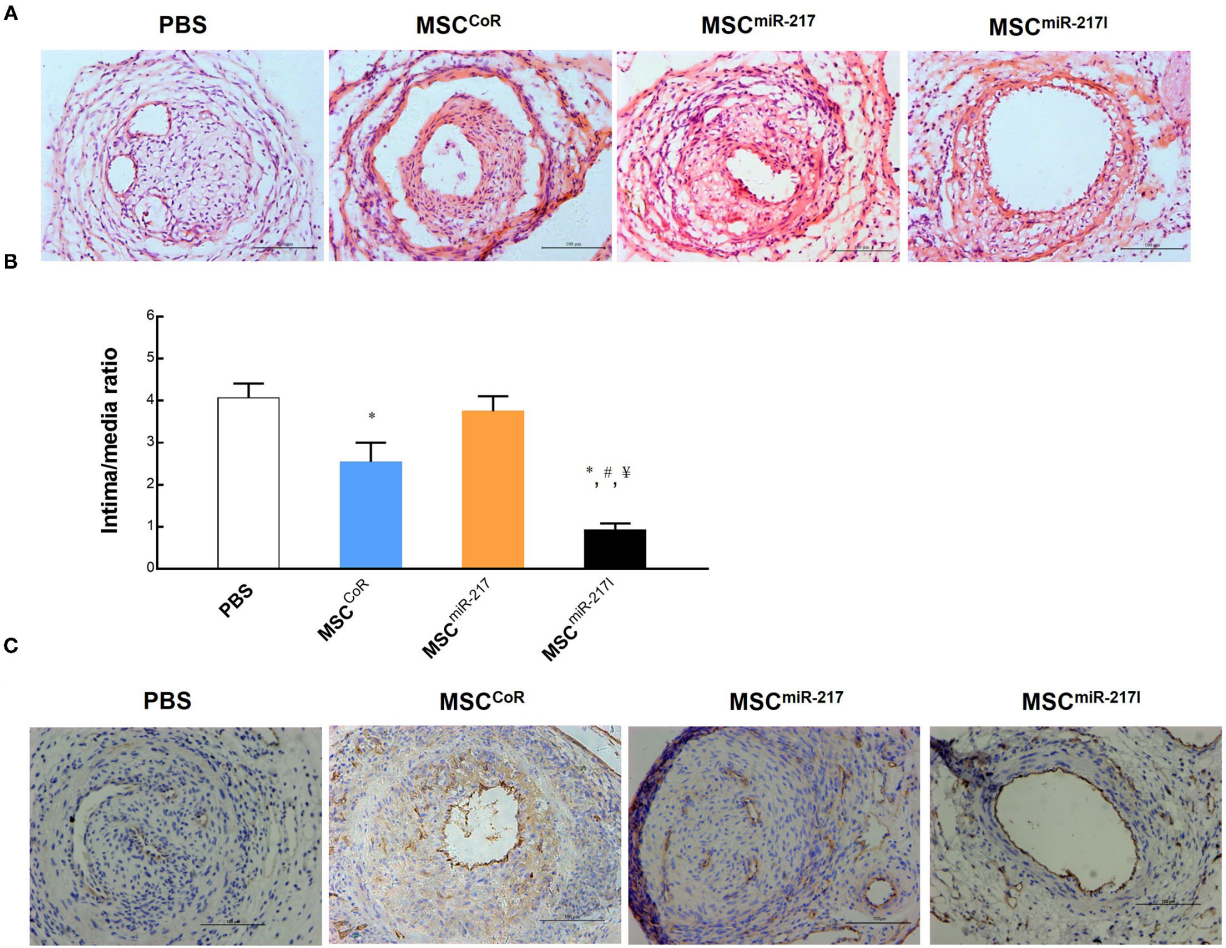
Evaluation of the therapeutic effect of MSC injection on re-endothelialization of the injured vasculature and the role of miRNA-217 in the injured artery repairment process. **(A)** Representative images of injured femoral arteries at 3 weeks after treatment with PBS, MSC^CoR^ (MSCs transfected with control siRNA), MSC^miR−217^, MSC^miR−217I^. Scale bars = 100 μm. **(B)** Analysis of the intima-to-media thickness ratio to reveal the cell treatment effect for neointima formation. *P < 0.05 vs. PBS, ^#^*P* < 0.05 vs. MSC^CoR^, ^¥^*P* < 0.05 vs. MSC^miR−217^. *n* = 9 each. **(C)** Immunohistochemistry analysis of injured vessels for CD31 endothelial marker expression at day 21 after the surgery and cell treatment.

To evaluate the protective effect of MSCs on IH, the tunica neointima-to-tunica media area thickness ratio was examined using Hematoxylin & Eosin (HE) staining across the sections. Compared to the PBS group, injection of MSCs transfected with control siRNA (CoR)-loaded NP (MSC^CoR^) significantly decreased the neointima-to-media thickness ratio (Figures 3A,B), indicating that MSCs successfully inhibited neointimal proliferation in the injured vascular endothelium. MiR-217 overexpression in MSCs abolished the therapeutic effect of the MSCs treatment on IH (Figure 3B), whereas MSCs transfected with miR-217 antagomir-loaded NPs (MSC^miR−217I^) significantly improved the therapeutic effect (Figure 3B), suggesting that miR-217 negatively regulates MSC-mediated recovery in neointimal hyperplasia. To evaluate efficiency of re-endothelialization of the injured artery, we stained the vessels with an endothelial cell marker CD31. Although each treatment group showed some degree of re-endothelialization, MSC^miR−217I^ treatment group exhibited the greatest number of CD31 positive cells surrounding the luminal surface of the repaired intima (Figure 3C).

### MiR-217 Inhibits Sirt-1 Expression, Causes EBs Malformation and Senescence in MSCs

**Figure 4:**
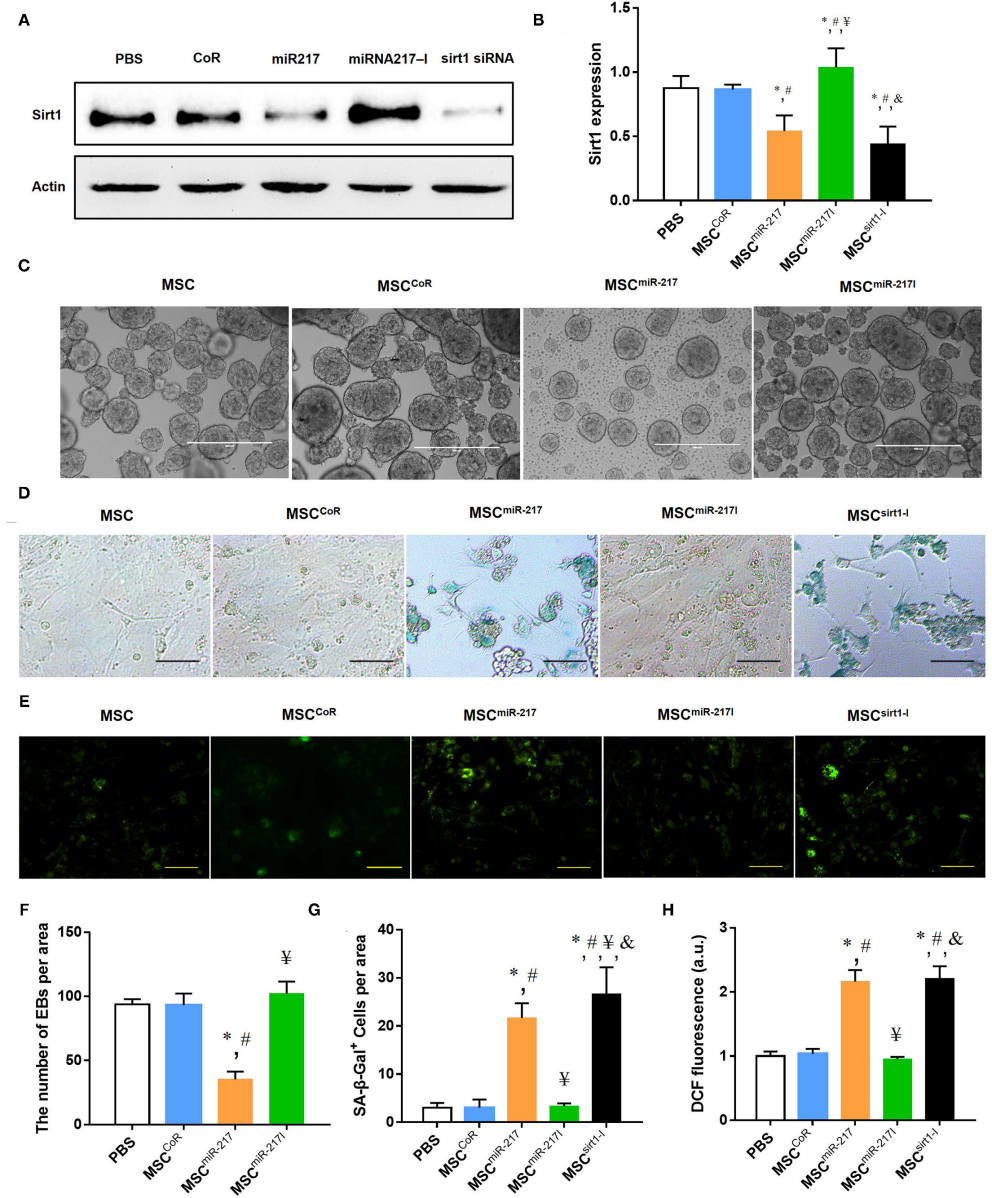
MiR-217 overexpression in MSCs reduces Sirt-1 expression, induces malformation of EBs, increased senescence and ROS activity. **(A)** A comparative analysis of the Sirt-1 expression in intact MSCs and MSC^CoR^, MSC^miR−217^, MSC^miR−217I^, MSC^Sirt1−I^ by Western blot. **(B)** Protein quantification by band densitometry in gels shown in panel **(A)**, *P < 0.05 vs. PBS, ^#^*P* < 0.05 vs. MSC^CoR^, ^¥^*P* < 0.05 vs. MSC^miR−217^, ^&^*P* < 0.05 vs. MSC^miR−217I^, *n* = 3. **(C)** Representative images of EB formation assay in each group. Scale bars = 500 μm. **(D)** Representative images of β-galactosidase (SA-β-gal) cell staining in each experimental group. Scale bars = 100 μm. **(E)** Fluorescence microscopic images of intracellular ROS production by DCF staining (green) in MSC cells. Scale bars = 100 μm. **(F–H)** Statistical analysis of EB quantification, SA-β-gal positive cells and DCF positive cells. *P < 0.05 vs. PBS, ^#^*P* < 0.05 vs. MSC^CoR^, ^¥^*P* < 0.05 vs. MSC^miR−217^, ^&^*P* < 0.05 vs. MSC^miR−217I^, *n* = 3.

Nicotinamide adenosine dinucleotide (NAD)-dependent deacetylase Sirt-1, also known as Sirtuin-1, is a critical factor that promotes angiogenesis and is one of the downstream targets of miR-217 (20). We hypothesized that the adverse effect of miR-217 on the MSC-mediated therapeutic activity on IH could result from downregulation of the Sirt-1 expression in MSCs. Indeed, transfection of miR-217 agomir into MSCs (miR-217 overexpression) significantly inhibited Sirt-1 expression, similar to the effect of Sirt-1 silencing achieved by the siRNA transfection of MSCs (Figures 4A,B). These results were consistent with previous reports on the inhibitory effect of miR-217 on Sirt-1 expression in tumor cells, liver cells and endothelial cells (20, 25, 26). In addition, miR-217 overexpression caused malformation of EBs (Figures 4C,F), which is an indicator of MSC malfunction and possibly impaired differentiation. Similar to the effect of Sirt-1 siRNA, inhibition of Sirt-1 expression by miR-217 overexpression caused a significant senescence in MSCs (Figures 4D,G). Furthermore, miR-217 overexpression could also increase reactive oxygen species (ROS) expression in MSCs (Figures 4E,H). These data suggested that the negative effect of miR-217 on the therapeutic role of MSCs resulted from increased senescence and impaired function of MSCs.

### Inhibition of MiR-217 Promotes MSC Adhesion and Re-endothelialization

**Figure 5:**
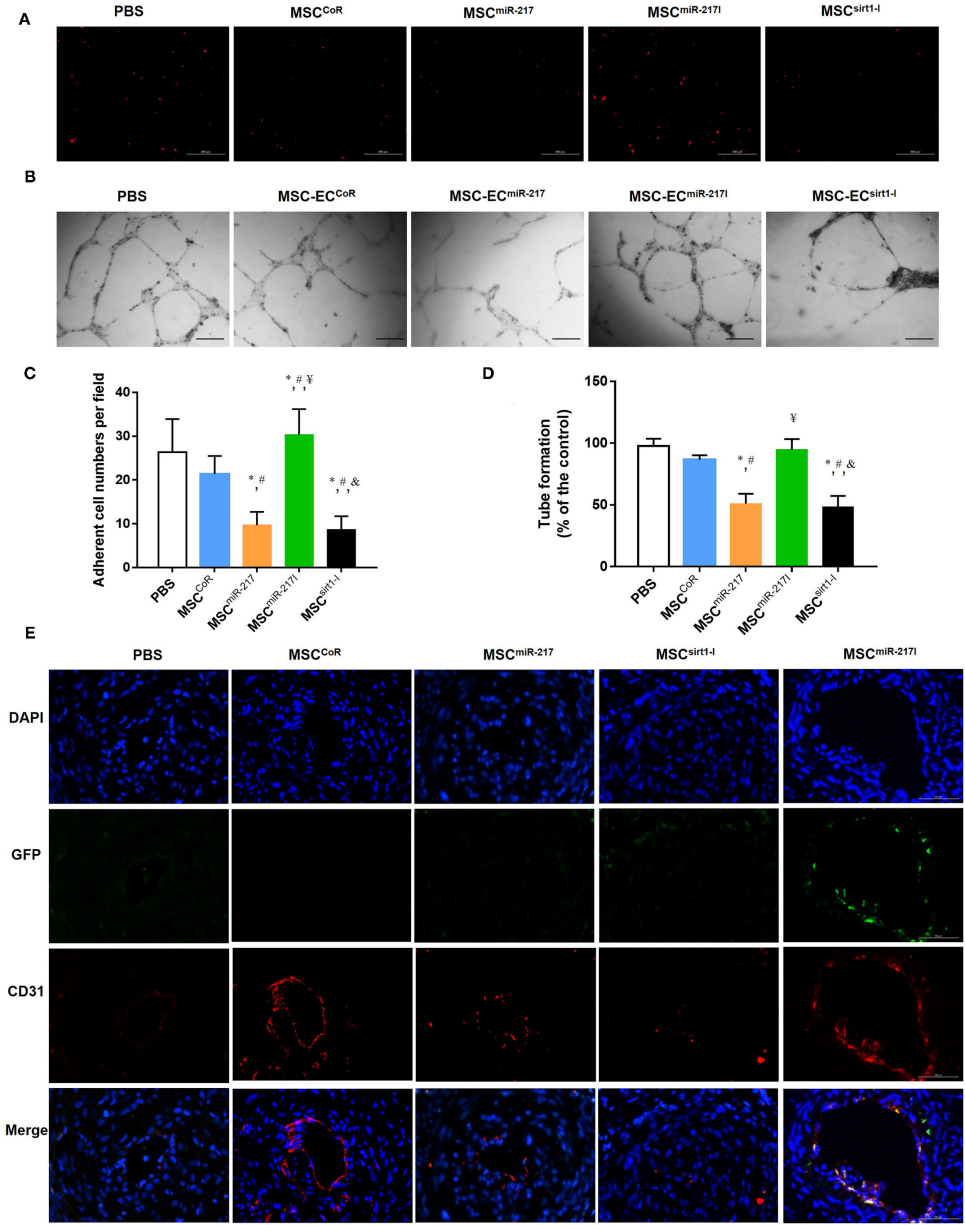
miR-217 inhibition can improve MSC treatment efficiency for IH by increasing the differentiation potential into endothelial cells at the injury site. **(A)** The adhesion properties of MSCs to the endothelial monolayer after treatment with PBS, MSC^CoR^, MSC^miR−217^, MSC^miR−217I^ or MSC^Sirt1−I^. **(B)** Assessment of MSC-EC tube formation properties in each treatment group. Scale bars = 200 μm. **(C)** Quantification of the adherent MSCs visualized for each sample in five randomly chosen fields (FOV) of duplicate chambers, *P < 0.05 vs. PBS, ^#^*P* < 0.05 vs. MSC^CoR^, ^¥^*P* < 0.05 vs. MSC^miR−217^, ^&^*P* < 0.05 vs. MSC^miR−217I^, *n* = 3. **(D)** Quantification of tube formation experiment. *P < 0.05 vs. PBS, ^#^*P* < 0.05 vs. MSC^CoR^, ^¥^*P* < 0.05 vs. MSC^miR−217^, ^&^*P* < 0.05 vs. MSC^miR−217I^, *n* = 3. **(E)** Immunofluorescent analysis revealed differentiation of engrafted GFP-labeled MSCs into endothelial cells. Paraffin sections of wound biopsies in each group were immunostained with anti-CD31 antibody and anti-GFP antibody. Fluorescence images reveal that MSCs expressing GFP (green) were incorporated into the vascular structures of the MSC-treated wounds and expressed CD31 endothelial marker (red). Nuclei were counterstained with DAPI (blue). The merged images evidence that the implanted GFP-labeled MSCs differentiated into CD31^+^ endothelial cells.

Previous studies suggested that the adhesion of MSCs to injured endothelial cells is required for MSCs to contribute to angiogenesis (27). We therefore performed a cell adhesion assay to determine if miR-217 inhibition can promote adhesion of MSCs to endothelial cells. As shown in Figures 5A,C, the basal adhesion activity did not differ significantly between MSCs and MSC^CoR^, while the adhesion activities of MSC^miR−217I^ increased dramatically. We next investigated the function of MSCs in a tube formation assay. As shown in Figures 5B,D, the tube length of MSC^miR−217I^ group enhanced dramatically, as compared to MSC^CoR^.

To evaluate the therapeutic effect of MSCs on neointima recovery *in vivo*, we analyzed vascular re-endothelialization at day 21 on the luminal surface of the injured arteries and assessed their integrity. As expected, more CD31^+^ cells were detected in the arterial lumens in cell treatment group as compared to PBS group (Figure 5E). However, re-endothelialization was significantly suppressed when applying MSCs with miR-217 overexpression. To track the injected cells, we also detected GFP fluorescence in the luminal surface. We assessed the ratio of GFP/CD31-double positive cells in injuried femoral artery at week 3 after surgery, double positive cells could be found in MSC^miR−217I^ group and could rarely be found in other cell treatment groups (Figure 5E). These results indicated that inhibition of miR-217 in MSCs accelerated re-endothelialization.

## Discussion

Intimal hyperplasia resulting from vascular injury is the main cause of vascular surgical reconstruction and endovascular treatment, which often leads to postoperative artery stenosis or occlusion. Current clinical approaches to reduce luminal stenosis induced by vascular injury include radiation, drug therapy, cell therapy and other strategies. However, the resulting therapeutic benefits are not satisfactory, while the cell therapy approach is hampered by limited cell sources.

MSCs hold promise for IH therapy owing to their self-renewal, vascular differentiation capabilities and paracrine properties (13) with some advantages lying in the straightforward cell isolation (from different sources) technique and ease of preservation. In addition, utilization of human MSCs raises no ethical concerns and harbors a limited risk of tumor (teratoma) development (14, 15). In this study, we demonstrated that intravenous administration of MSCs results in a significant therapeutic benefit for vascular injury repairment that occurs through re-endothelialization. GFP fluorescence of the transplanted MSCs allowed us to determine that MSCs could home to the vascular luminal surface of the injury site within 24 h, some of which survive for an extended time (3 weeks post MSC treatment). Co-localization of CD31 staining and GFP immunofluorescence indicated that at least some MSCs transdifferentiated into the vascular endothelial cells at the luminal surface of the injury site. Furthermore, the vascular intima-to-media thickness ratio was significantly reduced 3 weeks after MSCs administration. On the other hand, no obvious localization to the injury site was observed for the MSC^miR−217^, suggesting that miR-217 interfered with homing and/or differentiation of MSCs into endothelial lineage. The therapeutic effect of the MSC^miR−217^ was therefore significantly impaired by this inhibition. Our *in vitro* data showed that, miR-217 expression leads to knockdown of Sirt-1 protein expression, and increases senescence, ROS expression and malformation of EBs in the transfected MSCs. MiR-217 expression also inhibited the adhesion of MSCs and tube formation of MSC-ECs. These data suggested that miR-217 downregulates the expression of endogenous Sirt-1 in MSCs thereby hindering their natural process of homing to the vascular injury sites and basal function.

When MSCs were transfected with miR-217 inhibitor-loaded PLGA-NPs, they exhibited higher injury repairing capabilities. MSC^miR−217I^ showed a statistically significant difference in the ability to repair blood vessel damage relative to those of PBS and MSC^CoR^. Although MSC treatment could reduce intimal hyperplasia and promote re-endothelialization of the injured artery, almost no CD31^+^GFP^+^ ECs could be located at the luminal surfaces at week 3 after surgery. However, a large number of CD31^+^GFP^+^ ECs were found in the artery after MSC^miR−217I^ treatment, and the protective effect were significantly improved than the MSC^CoR^-treated mice, implying that miR-217 might affect MSC differentiation ability and have additional important biological roles in MSCs that warrant further studies.

Our data also suggested that miR-217 downregulates the expression of endogenous Sirt-1, thereby inducing cell aging and diminishing MSC differentiation capability. MiR-217 inhibition mediated by the miR-217 antagomir-loaded PLGA-NPs can significantly improve the efficacy of MSC-mediated IH treatment. In conclusion, our findings demonstrate a profound therapeutic benefit of MSCs on the endothelium recovery post vascular injury, which is negatively regulated by miR-217, offering a new potential IH treatment strategy.

## Data Availability Statement

The raw data supporting the conclusions of this article will be made available by the authors, without undue reservation.

## Ethics Statement

The animal study was reviewed and approved by the Ethics Committee of Wuhan Union Hospital.

## Author Contributions

HY, YHu, YHe, and HL contributed to design study. HY, YHu, YHe, JY, and HL analyzed data and prepared the manuscript. HY, YHu, YW, XH, SC, and JL contributed to do the experiments. HY and HL contributed to fund it. All authors contributed to the article and approved the submitted version.

## Funding

This work was supported by the National Natural Science Foundation of China (81500301 and 81600800).

## Conflict of Interest

The authors declare that the research was conducted in the absence of any commercial or financial relationships that could be construed as a potential conflict of interest.

## Publisher's Note

All claims expressed in this article are solely those of the authors and do not necessarily represent those of their affiliated organizations, or those of the publisher, the editors and the reviewers. Any product that may be evaluated in this article, or claim that may be made by its manufacturer, is not guaranteed or endorsed by the publisher.
