# Supplementary figures and images for: Sustained Release of MiR-217 Inhibitor by Nanoparticles Facilitates MSC-Mediated Attenuation of Neointimal Hyperplasia After Vascular Injury
Source: Front Cardiovasc Med. 2021 Oct 11;8:739107. doi: 10.3389/fcvm.2021.739107 (PMC8542691; doi:10.3389/fcvm.2021.739107)

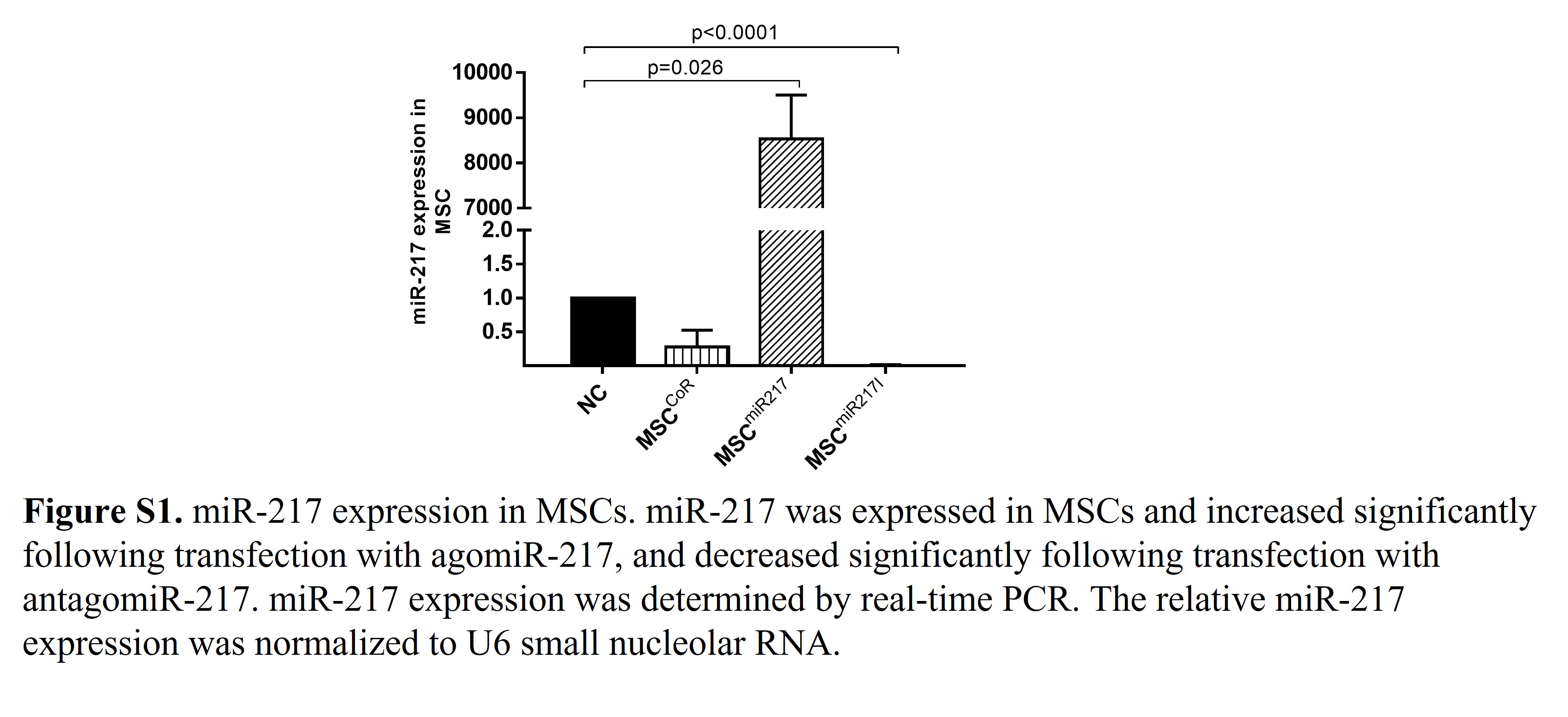

Supplement: Supplementary file 1 [file Image_1.JPEG]
